# Map Space Modeling Method Reflecting Safety Margin in Coastal Water Based on Electronic Chart for Path Planning

**DOI:** 10.3390/s23031723

**Published:** 2023-02-03

**Authors:** Da-un Jang, Joo-sung Kim

**Affiliations:** 1 Graduate School of Maritime Transportation System, Mokpo National Maritime University, 91, Haeyangdaehak-ro, Mokpo-si 58628, Jeollanam-do, Republic of Korea; 2 Division of Navigation Science, Mokpo National Maritime University, 91, Haeyangdaehak-ro, Mokpo-si 58628, Jeollanam-do, Republic of Korea

**Keywords:** map space, safety margin, concave hull, morphological, path planning

## Abstract

Map space composition is the first step in ship route planning. In this study, a map modeling method for path planning is proposed. This method incorporates the safety margin based on the theory of geographic space existing in coastal waters, maneuvering space according to ship characteristics, and the psychological buffer space of a ship navigator. First, the obstacle area was segmented using the binary method—a segmentation method—based on the international standard electronic chart image. Next, the margin space was incorporated through the morphological algorithm for the obstacle area. Finally, to minimize the space lost during the route search, the boundary simplification of the obstacle area was performed through the concave hull method. The experimental results of the proposed method resulted in a map that minimized the area lost due to obstacles. In addition, it was found that the distance and path-finding time were reduced compared to the conventional convex hull method. The study shows that the map modeling method is feasible, and that it can be applied to path planning.

## 1. Introduction

Motion-planning and path-planning algorithms are used to find the shortest distance between two points to avoid collisions of a moving object with impediments within the configuration space (C-space) [1]. C-space is composed of C-space obstacles, where a moving object collides with physical impediments, or certain designated links collide with each other [2]. Here, C-space refers to a set of locations, where the moving object is able to move, and a moving object is expressed as a point to resolve the issue of finding routes [3]. Therefore, planning a path in C-space, where the moving object is converted to a point, is simpler than planning the movement of a moving object in real space; furthermore, a more systematic and algorithm-based approach is ensured through the use of computer geometry [4,5]. Thus, through C-space, the geometric relevance between moving objects and obstacles can be clearly expressed in map space, and the user obtains a solution by properly mapping the path-planning algorithm to be solved in C-space.

Table 1 is a summary of a literature review related to map representation for path finding. To create a route for a moving object, Ref. [6] considered the UKC (Under Keel Clearance) and generated the region with the risk of standing as grids. Ref. [7] proposed a method that used the A* algorithm to find the shortest route for the environment with impediments according to the restrictive conditions of the vessel’s rotating radius. In addition, the generated route was validated through vessel simulation. Ref. [8] created a map space by processing captured images to create a route for rescue activities using a boat in a submerged environment. Ref. [9] proposed a method for generating routes by combining a quad tree and visibility graph in the coastal waters and setting water depth and weather information as consideration factors for path generation. Ref. [10] created a map space for route search based on satellite images. Obstacles were identified by image-processing satellite images, and identified obstacles were incorporated into the map space through the convex hull algorithm. Ref. [11] developed an algorithm for finding a free-space graph to determine the shortest distance through the boundary of the convex hull of an object. Ref. [12] proposed a method of generating a path that bypasses obstacles through the convex hull with an arbitrary type of obstacle boundary position and location information of the starting and arrival points of the moving object in the water space. Ref. [13] used the convex hull concept to propose a method of reducing the number of nodes of a complex mountainous terrain based on the stratified visibility graph that segments the map into certain altitudes.

Ship route planning can be detoured by simultaneously considering hazards from the vertical and horizontal directions existing in the sea space. In particular, routes in coastal waters have a relatively high risk; therefore, it is necessary to designate the boundary between the navigable waters and obstacle area on the sea; moreover, the ship operator should plan a route with a sufficient safety margin from the boundary of the obstacle area considering the characteristics of the ship. In the existing path generation study, obstacles existing in the water were incorporated into the map space, but there was no study that comprehensively considered the safety margin from the obstacle area. Therefore, in this study, in contrast to previous studies, in the process of constructing a map space for route generation, the safety margin from obstacles that simultaneously consider the topographic factors and the ship’s maneuvering characteristics was defined. In addition, the obstacle area within the map space should be configured in a geometric form, such that the moving object can explore the boundary of the obstacle. The existing convex hull technique, in the configuration of obstacle shapes, minimizes the area containing all points without an angle exceeding 180° between two adjacent edges. Accordingly, the boundary of the obstacle may be simplified in a complex environment, and the obstacle in the space may be comprehensively detected; however, the convex hull technique creates an area that contains all the points and, consequently, the shape of the original obstacle can be distorted. If the boundaries of the set point are distorted, in contrast to the existing form, the area in which the moving object can move may be lost, which can reduce route planning efficiency. The calculation of convex and concave hulls to sets of points in a two-dimensional space is a problem found in many different areas. Many known algorithms exist for calculating a convex hull, compared to those for calculating concave hulls [14]. Therefore, in this study, in contrast to the convex hull method, obstacles in the form of concave hulls, which do not distort the existing shape while simplifying complex boundaries, were modeled through image-processing techniques and computational geometric algorithms.

In other words, this study analyzed the physical topographic space of the sea, the control space according to the characteristics of the ship, and the psychological space considered by the ship operator, which was not incorporated in the previous map-space creation study, and defined the safety margin for the risk factors considered by the ship operator. The characteristics of the ship also define the shape of the obstacle region in the map space and propose an obstacle modeling that does not distort the existing shape while simplifying the complex boundaries. In addition, the purpose of this study is to incorporate the shape of the safety margin and obstacles in the image through morphological image-processing methods and geometric algorithms, as well as to generate a map space for safe and efficient route planning of ships.

The factors that constitute the safety margin were differentiated into vertical and horizontal directions. First, the safe water-depth margin for determining the safe-water boundary was set based on the water depth design presented by the Permanent International Association of Navigation Congresses (PIANC). Second, the safe distance for avoiding collision with impediments was established based on the ship domain model that considers the features of the vessel navigator and the tactical diameter as the turning circle. Based on the safe water-depth information, a hydrograph that incorporates the water areas inhibited for navigating was created. The hydrograph was transformed into a binary image that was treated with binarization in the form of a virtual grid. Here, the threshold image-segmentation technique was used to transform the electronic hydrograph image into the binary image. The boundary-extraction technique—an image-processing method—was used to express the boundary of the water inhibited for navigation from the impediments on the binary image. Additionally, the concave hull method was used to minimize the loss of the navigable area instead of the convex hull method. The convex hull method was frequently used in previous studies, among geometrical algorithms, for simplifying the boundary of the impediment [15]. Finally, to incorporate the safe distance for avoiding collision with impediments, the image-expansion method was used to create the C-space obstacles that enlarged the size of the impediments. Subsequently, the binary occupancy grid map was generated, and route generation through the PRM (Probabilistic Road Map) algorithm was chosen. The remainder of this paper is organized as follows: In Section 2, the background theory of this study is discussed. In Section 3, we will describe the methodology of C-space. In Section 4, we describe the simulation performed on the coast of South Korea and the simulation results. In Section 5, we draw conclusions and discuss recommendations for future studies.

## 2. Background Theory

### 2.1. Obstacles Area in Coastal Waters

**Figure 1:**
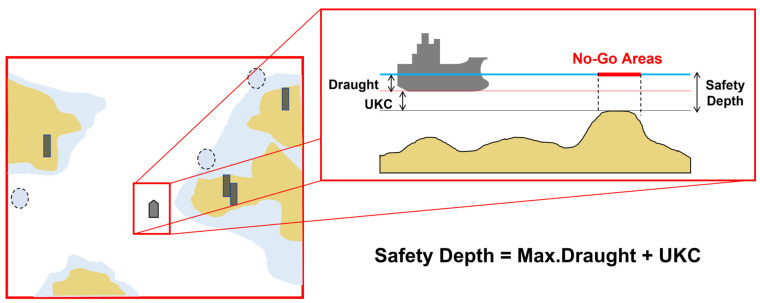
Chart showing the Safety Depth. The safe water depth indicates a value that includes the under keel clearance (UKC), which considers the navigation features of the vessel and marine environment to the maximum draught of a normal vessel.

The No-Go Area (NGA) indicates information on water areas that are not navigable according to the obstacle and water safety depth safe for the vessel as shown in Figure 1. In this study, the NGA set according to the safety depth of obstacles and vessels was designated as the obstacles area of C-space. In this study, the standard for safe water depth for designating obstacles area was based on the water depth design standard presented by PIANC.

In the PIANC (World Association for Waterborne Transport Infrastructure) [16] route guideline written by port and route design specialists, the concept design (CD) water-depth-design standard equation is provided. In PIANC, the standard equation for the water depth design is as follows:

(1) $$h_{cd}=F_{s}+S_{k}$$

where $F_{s}:\mathrm{Ship}\;\mathrm{Related}\;\mathrm{Factors}$; 

$S_{k\;}:\mathrm{The}\;\mathrm{equivalent}\;\mathrm{sinkage}\;\mathrm{of}\;\mathrm{the}\;\mathrm{ship}\;\mathrm{bilge}\;\mathrm{keel}$

Normally, $S_{k}\;$ is not used because the roll is not significant, such that $h_{cd}$ is equal to $F_{s}$. Therefore, the safe range in the vertical direction for impediments defined by this study is shown in Equation (2), and ship-related factors are shown in Table 2.

(2) $$h_{cd}=F_{s}$$

### 2.2. Safety Margin from Obstacles Area

The navigator must prioritize keeping the vessel within the margin space from the obstacles area when creating the coastal route; however, the margin space varies according to the type, size, and speed of the vessel including the experience of the navigator, and a firm standard is impossible. Therefore, this study referred to the margin space according to the ship’s domain model and turning circle of the vessel to maintain the minimum safety distance of the vessel from the obstacles. 

#### 2.2.1. Maneuvering Margin Space

**Figure 2:**
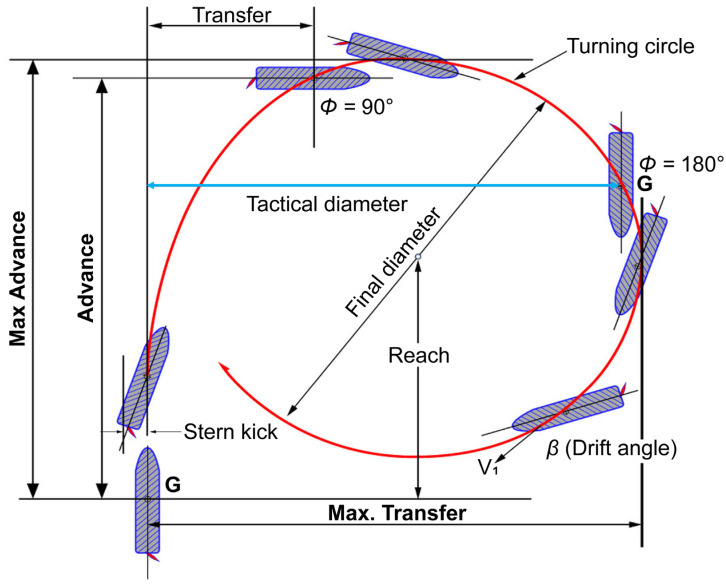
Turning circle components.

The navigating vessel will change the course or stop the engine in case of having to avoid risk factors. Normally, the stopping distance of the vessel is 10–15 times the vessel’s length. Therefore, in an emergency, the optimal decision for a vessel to avoid an impediment is veering through turning. Therefore, in coastal waters, vessels must maintain the minimum margin space possible to turn, so as not to collide with obstacles in an emergency. In this study, as shown in Figure 2, the minimum margin space required to avoid the obstacle area according to the turning ability of the ship was selected as the tactical diameter among the turning right factors. In addition, according to the performance standards of the IMO, the tactical diameter was set to 5×L (five times the length of the ship) [17].

#### 2.2.2. Bumper Margin Space

**Figure 3:**
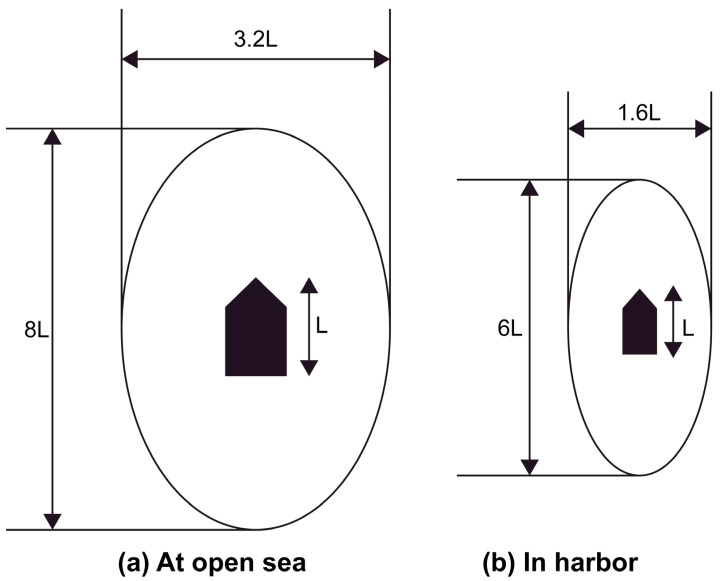
Ship’s bumper area. (**a**) In the bumper model, the water area that is navigable with a maximum speed of 10–16 knots and above is distinguished as the open sea. (**b**) The narrow waterway, in which the speed is reduced to 6–8 knots, is distinguished as the harbor.

Bumper space is defined as a safe distance to secure enough time to avoid obstacles in ship domain theory [18]. The bumper area was first proposed by Fuji et al. [19] and it is classified according to the navigation area, as shown in Figure 3. From the aspect of vessel navigation, the physical safety distance from the impediment is based on the lateral distance according to the vessel’s operations. Therefore, in this study, the safety distance was determined based on the bumper area of a short radius signifying the direction of the vessel’s lateral side in the open sea.

#### 2.2.3. Additional Safety Margin Space

In this study, the default value was taken as the minimum safety distance based on the vessel area model and the navigation capacity of the vessel mentioned above for the safety distance from impediments. If a higher level of safety was required owing to limited time or traffic concentration, an option was available for the user to set additional distances. 

### 2.3. Safety Margin Range

**Figure 4:**
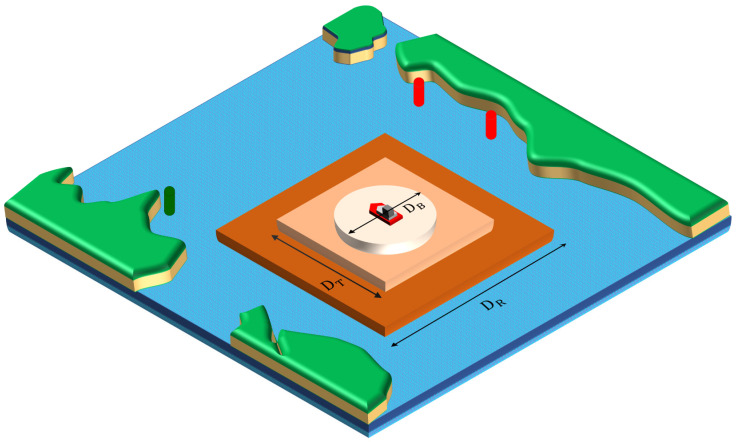
Description of margin space.

The safety margin range $\left(D_{R}\right)$ from the impediment defined by this study is shown in Equation (3) and Figure 4. Here, the safety range refers to the square with one side as the sum of the distance ($D_{Ship\;maneuverability},\;D_{T}$) according to the control capacity of the vessel, the distance the vessel navigator intends to maintain from the obstacles ($D_{Ship\;Bumper},\;D_{B}$), and additional user-dependent marginal distance ($D_{Additional},\;D_{Add}$) required; however, the safe distance refers to the range in the coastal water where the navigable area is not narrow or where a marine environment-specific, designated route exists.

(3) $$D_{R}={\left(D_{T}+D_{B}+D_{Add}\right)}^{2}$$

$D_{R}\;:\mathrm{Range}\;\mathrm{of}\;\mathrm{margin}\;\mathrm{space}$

$D_{T}\;:{\mathrm{Ship}}^{\prime }s\;\mathrm{maneuverability}\;\mathrm{distance}\;\left(5\;L:\;\mathrm{Tactical}\;\mathrm{diameter}\;\mathrm{of}\;\mathrm{IMO}\;\mathrm{regulation}\right)$

$D_{B}\;:\mathrm{Ship}\;\mathrm{bumper}\;\mathrm{distance}\left(3.2\;L:\;\mathrm{Short}\;\mathrm{radius}\;\mathrm{of}\;\mathrm{the}\;\mathrm{ship}\;\mathrm{domain}\right)$

$D_{Add}\;:\mathrm{Additional}\;\mathrm{distance}\;(\mathrm{If}\;\mathrm{user}\;\mathrm{needs}\;0\leq D_{Additional\;margin}<\mathrm{Half}\;\mathrm{width}\;\mathrm{of}\;\mathrm{waters})$

## 3. Materials and Methods

### 3.1. Process Flow

**Figure 5:**
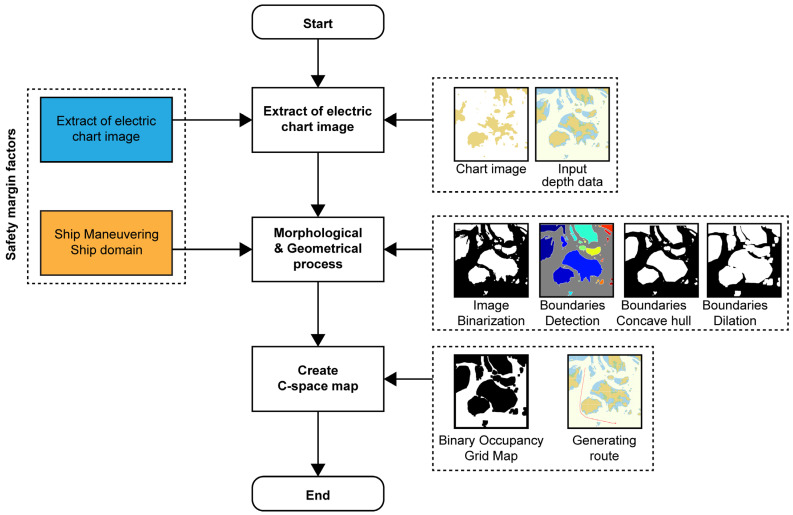
Process flow of the proposed map space method.

This study creates a C-space that incorporates the necessary factors for designating a route based on a hydrograph. The final purpose of this study is to generate a safe and economic route through the map space (C-space). The procedure of this study is shown in the flow chart illustrated in Figure 5.

### 3.2. Set of the Electronic Chart Range

A hydrograph refers to a specially designed blueprint that indicates dangerous impediments, route signs, form and features of the coast, the height, bottom mud, and water depth [20]. To ensure safe navigation, a ship operator draws a route considering the depth of water, the topography of the seabed, aerial obstacles incorporating the height of the ship, the shape and characteristics of the coast, route signs and dangerous obstacles, etc. Restricting the range of the electronic navigation chart is necessary for creating a map space for certain coastal water. Therefore, in this study, the range was restricted by maintaining a distance in the direction of the coordinates with a certain coordinate as the starting point. Given a starting point, initial bearing, and distance, this will calculate the destination point and final bearing traveling along a great circle arc. The equation for setting the range is shown in Equation (4).

(4) $$\varphi_{1}=asin\left(sin\varphi_{1}\cdot cos\delta +cos\varphi_{1}\cdot sin\delta \cdot cos\theta \right)\;\lambda_{2}=\lambda_{1}+atan2\left(sin\theta \cdot sin\delta \cdot cos\varphi_{1},\;cos\delta -sin\varphi_{1}\cdot sin\varphi_{2}\right)$$

φ :Latitude

λ :Longitude

$\theta \;:\mathrm{Bearing}\;\left(\mathrm{Clockwise}\;\mathrm{from}\;\mathrm{north}\right)$

$\delta \;:\mathrm{Angular}\;\mathrm{distance}\frac{d}{R}\left(d:\mathrm{Distance}\;\mathrm{travelled},\;R:\mathrm{The}\;{\mathrm{earth}}^{\prime }s\;\mathrm{radius}\right)\;$

### 3.3. Set the Safety Depth Boundaries on the Electronic Navigational Chart

**Figure 6:**
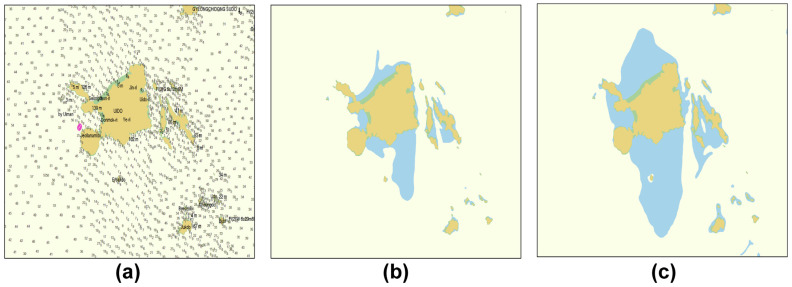
Apply safety depth on the electronic navigational chart image. (**a**) Set of the electronic navigational chart. (**b**) Set the safety depth to 10 m (**c**) and set the safety depth to 5 m.

In general, the route planning designed by the ship navigator is created with a paper and electronic hydrograph, and the hydrograph is a specially designed drawing for indicating the water depth, type of seabed, height, form and features of the coast, and route sign. In this study, the electronic hydrograph image for navigation, as shown in Figure 6, was used to model the route generation space according to the marine environment and vessel features. The electronic chart used in this study was based on the electronic navigational chart produced by the Korea Hydrographic and Oceanographic Agency (KHOA), in accordance with the S-101 standard. Furthermore, the safe water depth was determined according to the water depth design standard of PIANC on the electronic hydrograph image. Using the depth parameter control function on the electronic hydrograph image, the safe water depth boundary was incorporated on the map, as shown in Figure 6b,c.

### 3.4. Segmentation of Obstacle Area on the Chart Image

Image segmentation is a technique widely used in digital image processing and its analysis divides an image into multiple parts or regions based on the characteristics of pixels in the image. The approach of image segmentation detects the similarity between the regions of an image, which includes region expansion, clustering, and binarization. In this study, image segmentation was performed through a binary processing method that divides an image into several segments and processes the entire image. It consists of a process of converting one image into pixel regions represented by a mask or labeled image. A binary image means an image expressed only in black and white with 1 bit per pixel and is performed through threshold processing. The threshold value (T) technique is a throughout fixed binarization (b) processing technique that transforms white (=1) or black (=0) image pixels as shown in Equation (5).

(5) $$b\left(j,i\right)=\left\{\begin{array}{c} 1,\;\;f\left(j,i\right)\;\geq T\\ 0,\;\;f\left(j,i\right)<T\; \end{array}\right.$$

**Figure 7:**
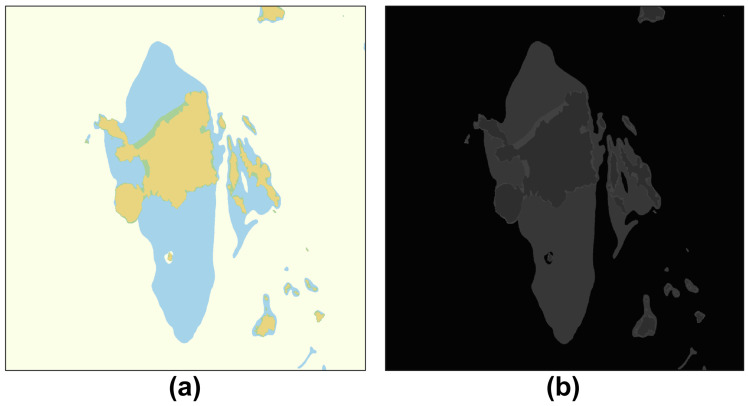
Convert chart image to grayscale image. (**a**) Extracted electronic chart image, (**b**) Convert to a grayscale image.

First, the two-dimensional RGB color image of the electronic navigational chart image incorporating the safety depth information is converted into a grayscale image. A grayscale image is an image that can express 256 levels of light and shade and transmits luminous intensity information representing the amount of light in each pixel. Grayscale image conversion creates a gray colormap by converting the colormap to NTSC coordinates and setting the hue and chroma components to zero. Thereafter, the index of the image is converted to the corresponding grayscale value of the gray colormap, as shown in Figure 7.

**Figure 8:**
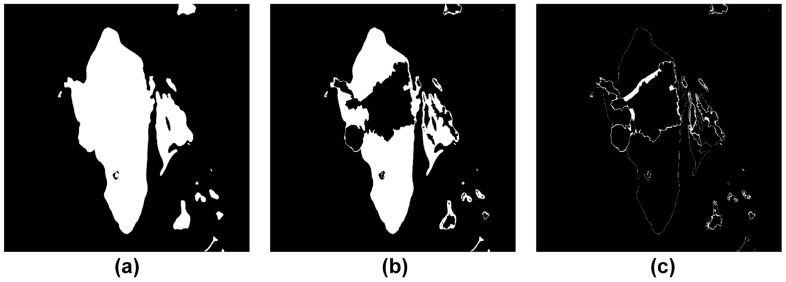
Apply binary segmentation by the Otsu algorithm. (**a**) Set T=10, (**b**) Set T=50. (**c**) and set T=100.

Otsu’s binarization algorithm [21] was performed on the 2D grayscale image to create a binarized image divided into black and white. Otsu’s algorithm divides pixels into two classes by randomly setting a threshold value, repeats the task of obtaining the intensity distribution of the two classes, and selects the threshold value when the intensity distribution of the two classes is the most uniform among all cases. Therefore, in the Otsu algorithm, when an image is segmented based on a specific Threshold T value, the T value minimizing the weighted sum of the variances of both images is repeatedly calculated and found, as shown in Figure 8. 

The Otsu technique sets the threshold value by maximizing $\sigma^{2}{}_{B},$ the dispersion between clusters, or minimizing the $\sigma^{2}{}_{W},$ an internal dispersion of the cluster $C_{1}=\left[0,\;c\right]$ and $C_{2}=\left[c+1,\;L-1\right]$ segmented by a random brightness c for the image composed by the degree of brightness of $\left[0,\;L-1\right]$. Here, the total variance $\sigma^{2}{}_{T}$ satisfies the relation of $\sigma^{2}{}_{T}=\sigma^{2}{}_{W}+\sigma^{2}{}_{B}$, so that the grayscale image I given by an arbitrary intensity c is, when divided by $C_{1}$, $C_{2}$, the optimal threshold is calculated as in Equation (6).

(6) $$c=argmin_{0\leq c\leq L-1}\left[\omega_{1}\sigma_{1}^{2}+\omega_{2}\sigma_{2}^{2}\right]$$

where $argimin\left(arguments\;of\;min\right)$ means that the points in the domain that make a function minimal are called elements or parameters. In other words, it is the value of t that makes the variance minimal.

$$\sigma_{1}^{2}=\sum_{i=0}^{c}p_{i}{\left(i-\mu_{1}\right)}^{2}/\omega_{1},\;\sigma_{2}^{2}=\sum_{i=c+1}^{L-1}p_{i}{\left(i-\mu_{2}\right)}^{2}/\omega_{2},\;\mu_{1}=\frac{\sum_{i=0}^{c}i\cdot p_{i}}{\omega_{1}},\;\mu_{2}=\sum_{i=c+1}^{L-1}i\cdot p_{i}/\omega_{2},\;\omega_{1}=\sum_{i=0}^{c}p_{i,\;}\omega_{2}=\sum_{i=c+1}^{L-1}p_{i},$$

### 3.5. Extract of Obstacle Boundaries on the Binary Image

**Figure 9:**
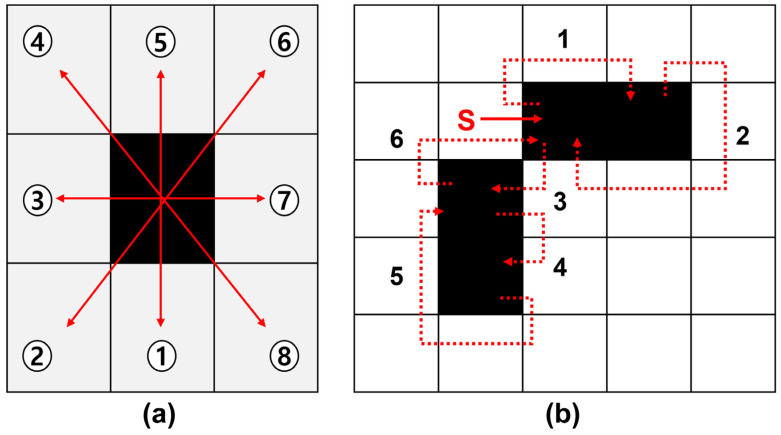
Contour following the sequence of the MNT. (**a**) Boundary tracking method. (**b**) Tracking the boundary of the obstacle area in the binary image.

The boundary coordinates of the object must be extracted from the pixel image consisting of [0, 1] to simplify the complex boundary of the obstacles in the binary image. The boundary tracking technique was used in the area boundary of the binarization image to extract the external boundary coordinate information of the obstacles from the binary image. The edge-tracking algorithm is an algorithm that tracks the outline of a specific object in an image. Considering line segment information and direction change information can be obtained according to the direction information of pixels constituting the boundary of an object, it is very useful when finding feature points of an object. In this study, the Moore-Neighbor tracing algorithm [22] was used to track the boundary of the obstacles area in the binary image. The Moore-Neighbor tracking (MNT) algorithm tracks specific pixels in eight clockwise directions (D→DL→L→UL→U→UR→R→DR), as shown in Figure 9. Equivalently, two pixels adjacent to a particular pixel belong to the same object if they are connected horizontally, vertically, or diagonally. As shown in the starting point in Figure 9b, when the tracker encounters a pixel in the obstacle area, it searches in a clockwise direction to find an adjacent pixel. As the tracking continues, the algorithm ends when the starting pixel of the obstacles and the entry direction are the same.

**Figure 10:**
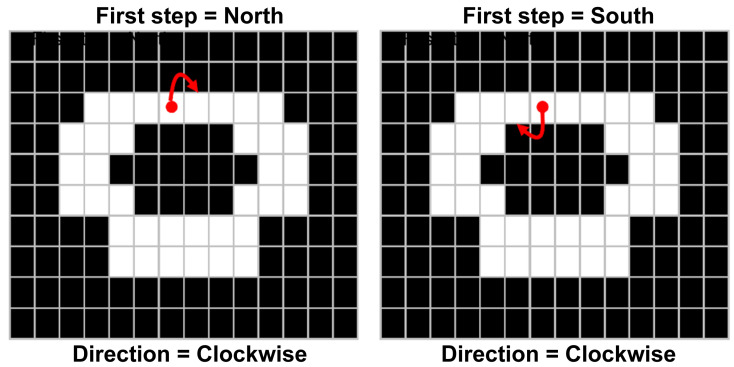
Effect of direction parameters on boundary tracking. In this figure, red color arrow means direction of tracing.

Figure 10 depicts the pixels traced when the first step is set as the north direction, and the south direction in starting pixels set as the eight connections. Some objects include a hole, and depending on the selection of certain pixels as starting pixels, the outer or inner boundary of the object could be traced.

### 3.6. Generated Obstacle Boundaries

**Figure 11:**
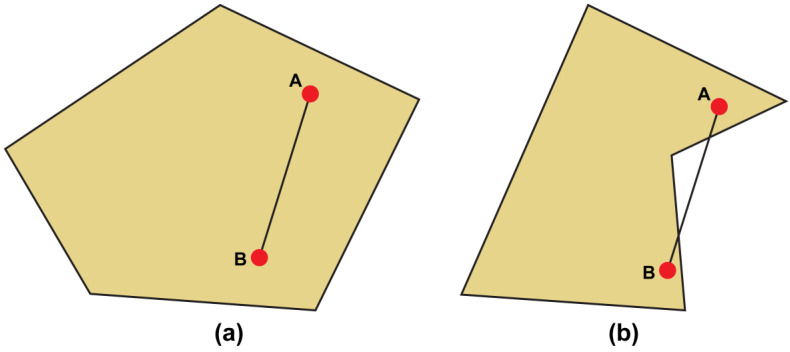
(**a**) Convex and (**b**) concave polygons.

Finding a concave boundary for the set of some points, one geometrical algorithm is applicable in various circumstances. Convex or concave algorithms are used to find the minimum area that encompasses the set point or defines the shape of a polygon. Figure 11a presents a convex polygon, and the convex polygon is included in the space where two randomly selected points are linked. Figure 11b presents a concave polygon, from which a line connecting two points that pass the outer boundary of the shape exists inevitably.

Consequently, the method of creating a polygon through the points in a random point set is referred to as the convex hull and concave hull. If the convex concept exists between all points within a certain area, the set of all points within the area can be referred to as the convex set. The convex set is defined by Equation (7), and if any set C does not hold, Equation (7) is called a concave hull equation.

(7) $$x=ax_{1}+\left(1-a\right)x_{2},a\in \left[0,1\right]\;x_{1},x_{2}\in C,\;a\in \left[0,1\right]\to ax_{1}+\left(1-a_{x_{2}}\right)\in C$$

**Figure 12:**
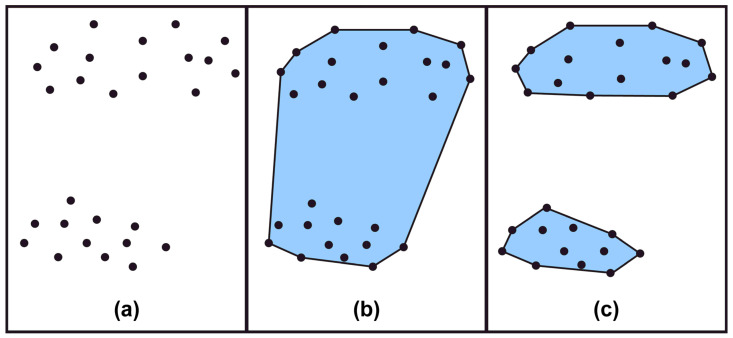
Convex and concave hull algorithms. (**a**) A set of random points. (**b**) The convex hull is connected and shows a polygon (**c**) The concave hull shows two polygons.

Figure 12 shows the results of forming a polygon through convex and concave hull methods for a random point set. As shown in Figure 12a, for the set of points distributed in two distinguished areas, the convex hull algorithm constitutes them into one area through the polygon and generates a loss area. However, the concave hull algorithm, as shown in Figure 12c, can be segmented into two areas without an area of loss. The boundary between water and land in marine environments is formed in the shape of complex curves instead of a straight line, and the vessel navigator simplifies this to indicate this on the hydrograph for easy recognition of the complex boundaries during sailing. Some previous studies simplified the boundary of the obstacles composed of complex curves for route searching through the convex hull algorithm. The advantage of the convex hull was the ability of the moving object to move at the shortest possible distance along the boundary points of the impediment. However, in the case of the convex hull algorithm, because it is a method of constructing a boundary by simply connecting edges, there is a limit to implementing the shape of an obstacle or contour line in the coastal waters constituting a complex coastline. Furthermore, during the process of creating the boundary of the obstacle, there is a possibility that the movable areas may be lost. Consequently, unnecessary bypass routes may occur for the moving object. Therefore, in this study, obstacle area boundaries were created through a concave hull algorithm using an alpha (α) shape to prevent loss areas from occurring due to obstacles boundary generation.

The alpha shape, a geometric calculation method, is a convenient way to represent relatively simple boundaries, such as convex and concave hulls, constructing a bounding area or volume that encloses a set of 2D or 3D points [23]. The alpha shape draws a circle with radius α connecting the contours of two randomly selected points to form a 2D or 3D object surrounded by several circles. In point clouds for complex objects, sophisticated shapes can be implemented in contrast to the convex hull method, which simply connects the points on the outside [24]. In addition, a non-block area can be created by tightly fitting the shape near the point or loosening it. In addition, it is possible to add or remove a point or to not indicate a hole. 

In this study, the boundary of the coastal water’s obstacle area was constructed using the concave hull algorithm based on the alpha shape. By manipulating the alpha shape scaling factor, the boundary of the obstacle area becomes convex or concave [25].

First, let us define a generalized disk of radius 1/alpha to be the following:

- If alpha > 0, it is an ordinary closed disk of radius 1/alpha;
- If alpha = 0, it is a halfplane;
- If alpha < 0, it is the complement of a closed disk of radius −1/alpha.

Then, given a set of points and a specific value for alpha, we construct the alpha shape graph in the following manner:

- For each point $P_{i}$ in our point set, we create a vertex $V_{i}$.
- We create an edge between two vertices, $V_{i}$. and $V_{j}$, whenever there exists a generalized disk of radius 1/alpha containing the entire point set, and which has the property that $P_{i}$ and $P_{j}$ lie on its boundary.
- If α = 0, then the alpha shape associated with the finite point set is its ordinary convex hull.

**Figure 13:**
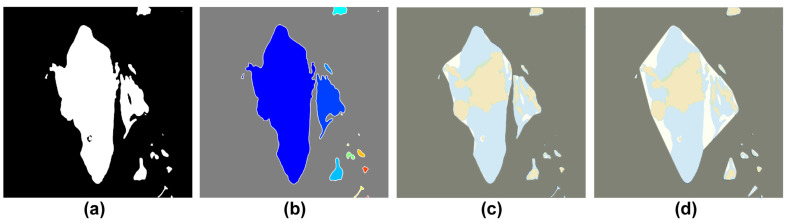
Construction of the obstacles area boundary. (**a**) Binary image from electronic chart image. (**b**) Tracking of obstacles boundaries. (**c**) Shape of the boundaries using the convex hull (**d**) Shape of the boundaries using concave hull through the alpha shape.

Figure 13 shows the boundary designation process of the obstacle area proposed in this study. Figure 13b is the step of tracking the boundary points of the obstacle area, and Figure 13c,d show convex and concave hulls because of boundary simplification, respectively.

### 3.7. The Obstacle Boundaries Processing Incorporating the Safety Margin

**Figure 14:**
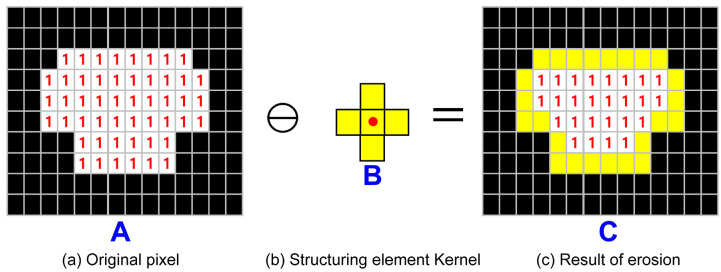
Erosion process on image data. (**a**) Blue letter A is original pixel. And it shows the black pixel represents zero and the white pixel represents one. (**b**) Blue letter B is cross-shape structuring element kernel. (**c**) Blue letter C is result of morphological erosion.

C-space refers to a set of positions where a moving object can move. In C-space, a moving object is expressed as a point to solve the problem of path search. Therefore, in the map configuration, the geometric relationship between the object and obstacle can be clearly expressed by adjusting the obstacles constituting the surrounding environment according to the characteristics of the moving object. In this study, the expansion method of the morphological algorithm was used to incorporate the safety margin in the obstacle area in the C-space. Morphology algorithm [26] is a method used in image processing, and it is used to remove noise, fill holes, and link disconnected lines according to the given form in a black-and-white binary image. The most typical methods among the morphological algorithms are erosion and dilation. First, the erosion method refers to the computation that trims the image. The binary morphologies (A ϴ B) of *A* due to *B* from Figure 14 are defined by the same set of computations as Equation (8).

(8) $$A\;ϴ\;B=\{z|\left(B\right)z\subseteq A\}$$

For the erosion method, the structuring element kernel composed of zero and one is necessary. The structuring element kernel can be used with a square, ellipsis, and cross shape depending on the shape of the filled one. Figure 14 shows the erosion method of the process of the cross-shape structuring element kernel. In Figure 14a, the black pixel represents zero and the white pixel represents one. Figure 14b is the cross-shape structuring element kernel, and the yellow pixels including the center are all composed of one. Here, the cross-shaped structuring element kernel center (red point) scans the white pixel of Figure 14a. During the scanning of each block, the structuring element kernel becomes zero when it does not entirely overlap the white pixel. If it overlaps entirely, it is maintained as one. The results of erosion computation are shown in Figure 14c, where the reduction of the area that the number “1” occupies can be known.

**Figure 15:**
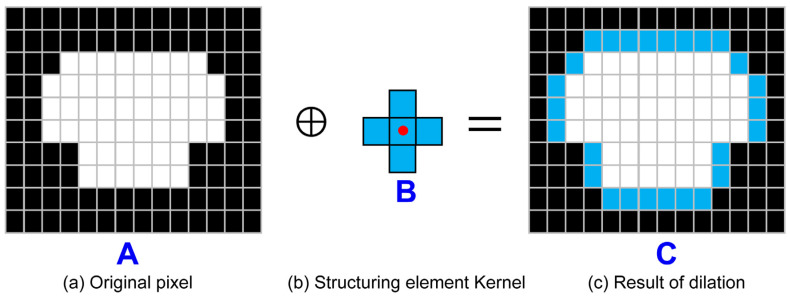
Dilation process on image data. (**a**) Blue letter A is original pixel image. (**b**) Blue letter B is cross-shape structuring element kernel. (**c**) Blue letter C is result of morphological dilation.

In contrast to the erosion method, the dilation method applies computation that expands the surrounding of the object. In Figure 15, the binary image dilation (A⊕B) of A due to B is defined as the computation set, such as Equation (9). Here, $\hat{B}$ denotes the symmetry of the structuring element kernel B.

(9) $$A⊕B=\{z|(\hat{B})z\cap A\neq \emptyset \}$$

The erosion method was transformed into zero if the structuring element kernel did not entirely overlap the area filled with ones in the input image; however, in the dilation method, it was changed to one if it did not entirely overlap. Figure 15 shows the image dilation process through the structuring element kernel: Figure 15a shows an original pixel image and Figure 15b shows a cross-shape structuring element kernel. Figure 15c shows an expansion of the original image as C. This study uses the dilation method among the morphology computations to create the c-obstacle after image processing, which incorporates the safety range of the vessel to the impediment.

**Figure 16:**
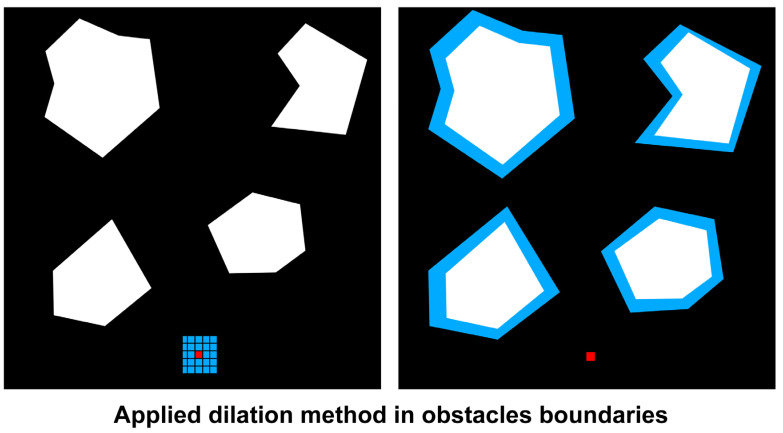
Applied dilation method in obstacles boundaries.

As shown in Figure 16, c-obstacles were constructed by expanding the obstacle area by the size of the minimum free space of the ship. Through this dilation method, it is possible to create a safe path as much as the minimum free space through an obstacle area larger than the existing area.

## 4. Experiment Results and Discussions

**Figure 17:**
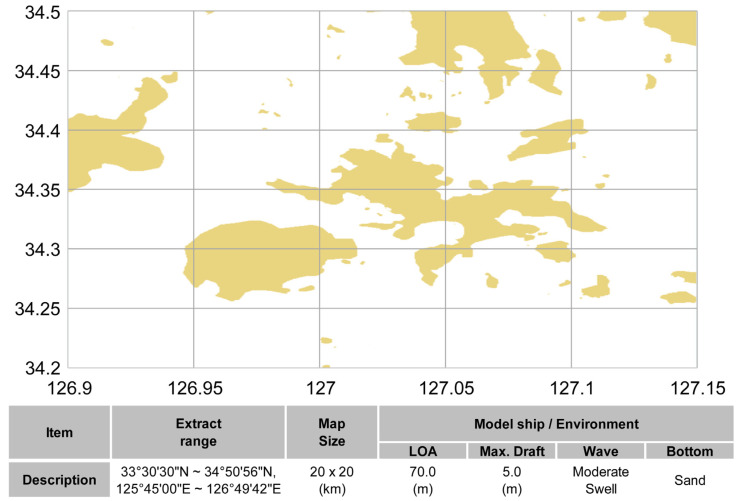
Description of simulation.

This study aims to develop a map space for creating a safe and economic path that suits the features of a vessel. Therefore, the simulation required a vessel model for navigating the sea areas. A model vessel for simulation should be able to represent the sizes of various ships on the coast. In this study, a standard vessel was selected as the simulation model, which was used to assess the marine traffic congestion in Korean coastal waters [27]. A standard ship is a ship of 70 m in length and a gross tonnage of 1000 tons calculated through a correlation function between ship length and gross tonnage [28]. In addition, the target sea area for the simulation was the southern sea of the Korean Peninsula near numerous islands and low water depth. Figure 17 shows the target sea area and specifications for performing the simulation.

**Figure 18:**
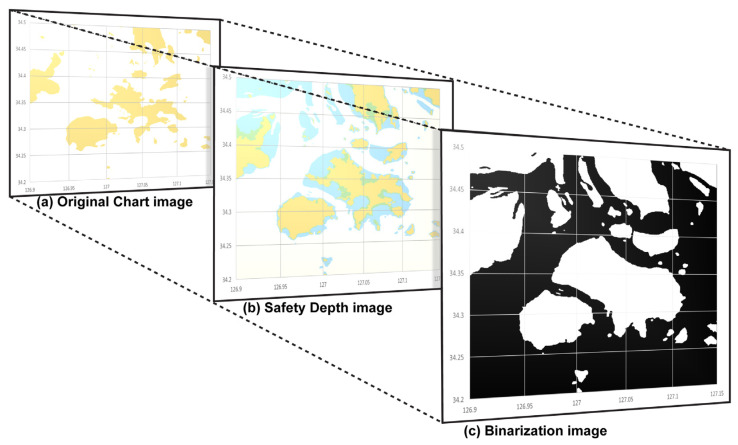
Binarization of the chart image including the safety depth (**a**). The original chart image for simulation (**b**) Set No-Go Area boundaries using the safety depth and obstacles data (**c**) Binarization of the chart image for simulation.

First, the safe water depth according to the draught of the model vessel in the target sea area was determined based on the PIANC standard, and the result value ($h_{cd}=7.0\;m)$ was incorporated in the hydrograph in Figure 18a. The boundary of the water area prohibited for navigation based on the impediment object and safe water depth was generated, as shown in Figure 18b. The hydrograph image was transformed into a binary image in which safe water depth data was incorporated to constitute the map space for generating routes, and the results are as shown in Figure 18c.

**Figure 19:**
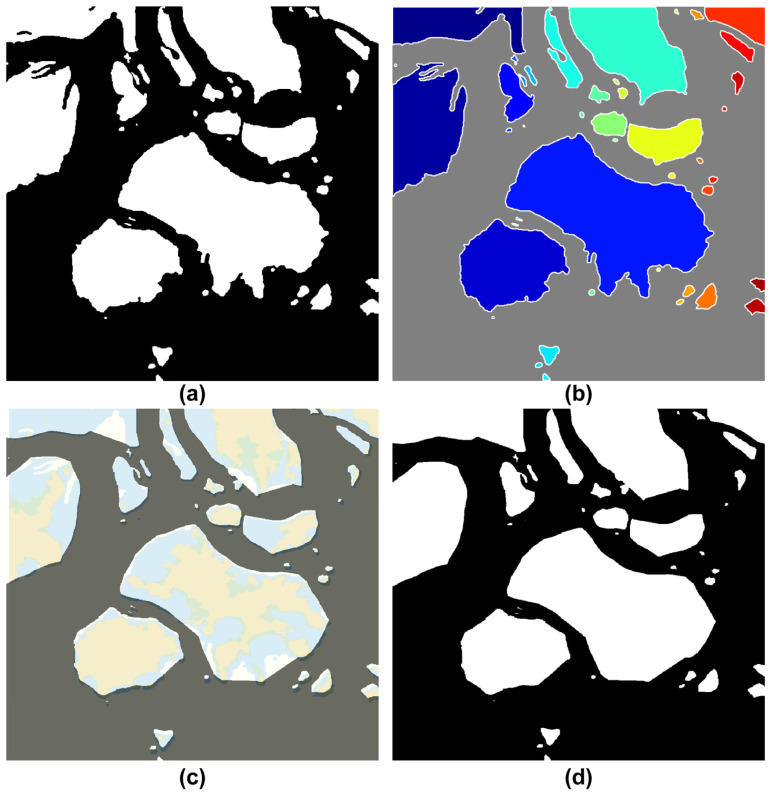
Simplified obstacle boundary process (**a**) Binarization map including the safety depth data. (**b**) Detection of obstacle boundaries. (**c**) Concave hull method (Shrink Factor: 0.9). (**d**) Simplified obstacle boundaries.

**Figure 20:**
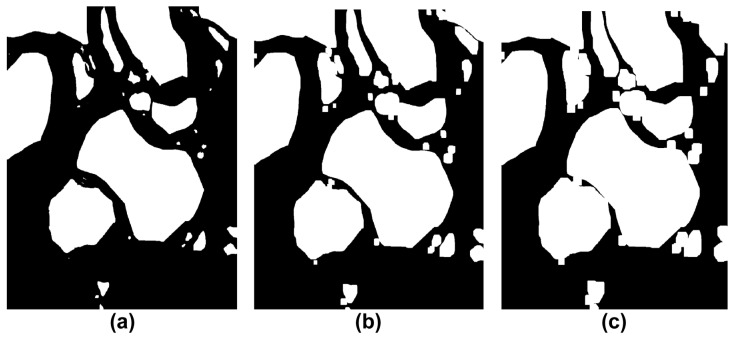
Comparison of obstacles’ dilation results on the concave map. (**a**) Binary map of the simulation area. (**b**) Results of dilation on the concave map $\left(D_{r}=574\;m\right)$. (**c**) Results of dilation on the convex map $\left(D_{r}=1074\;m\right)$.

This study simplified the concave hull method for the boundary of the non-navigable water areas composed of impediments and low-water-depth regions in the target sea area of the simulation. Figure 19 shows the concave hull method-application process and impediment-boundary tracing method of the binarization image. First, Figure 19b shows the extraction of coordinates from the binary image in Figure 19a through the tracing method. The 0.9 factor generated the optimal boundary plane, where the shrink factor range ($0\;\leq F_{s}\leq 1$) was as shown in Figure 19c. Figure 19d shows the results obtained after simplifying the boundary through the concave hull method. Additionally, Figure 20 shows the results obtained from incrementally increasing the safety range for the vessel by applying the dilation technique on the simplified boundary. 

Figure 20a shows the results of the concave hull for the hydrograph that accounts for the safe water depth. Figure 20b shows the results of solely considering the minimum safe distance from the impediment. Figure 20c shows the results that were obtained by assuming a minimum safe distance of 500 m. If an additional margin value is added to Figure 20c, the size of the impediment expands, and a C-space is formed in a similar shape to the area of the convex hull as the navigable area which was lost, in terms of the width of the route.

**Figure 21:**
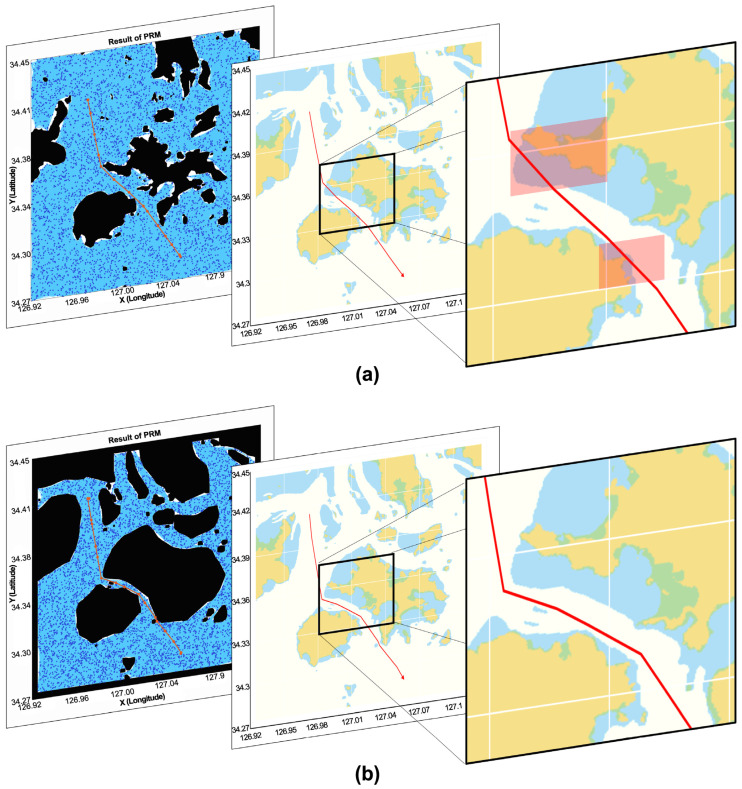
Route generation results when (**a**) the safety factor was not considered and (**b**) when the safety factor was considered.

The obstacles area generation method, conducted through the existing convex hull, and the map space composition results, proposed through the concave hull, were compared using the PRM algorithm among the route-generation algorithms. A PRM [29,30] is a multiple-query sampling method that is an entire-area route-planning method for generating the optimal route. A PRM generates a route efficiently within a short time in the complex and high-dimensional space, and it is applied in various sectors including robot engineering and autonomous driving [31]. The PRM generated a roadmap that connected the nodes without collisions, which are randomly selected from a certain starting point. The nodes of a certain starting point and a randomly selected point were linked, and the closest node in the destination point was linked to the algorithm that searched for the route to the destination point. Figure 21 shows an image comparing the results obtained when the safety margin was considered and when it was not considered. Figure 21a shows the results of generating a route in the map area where the safety distance was not incorporated, and the generated route was limited by the water-depth area, which is why no sufficient safety could be ensured for the impediment. Figure 21b shows the results of generating the route on the map area that considered the minimum safety distance from the impediments and the safe-water-depth data of the vessel, where no overlapping areas existed within the risk areas.

**Figure 22:**
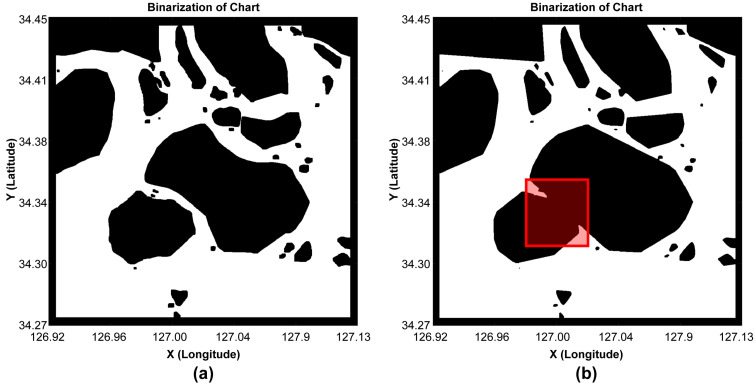
Comparison of the binary occupancy maps. (**a**) Concave hull map. (**b**) Convex hull map.

Additionally, to validate the efficiency of the map area based on the concave hull method, the convex hull method mainly used to simplify the impediments was comparatively analyzed based on the route generation results. First, the map-area generation results for generating routes showed that the boundary was more simplified in the convex hull than in the concave hull, where the safe water depth and safety distance were incorporated in the existing chart image, but Figure 22 shows that there were areas of loss. 

**Figure 23:**
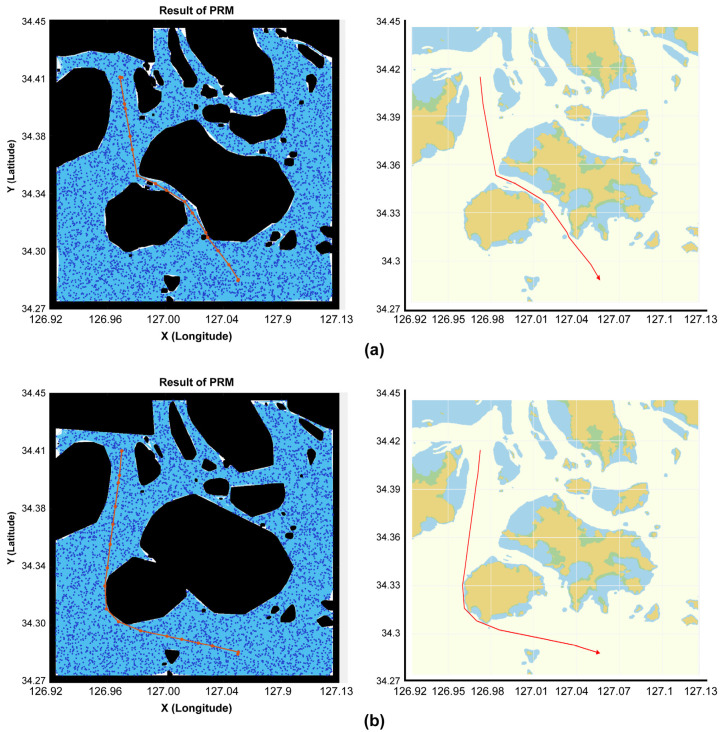
Route generation results. (**a**) Concave hull method. (**b**) Convex hull method.

The comparison of the results of the concave hull method proposed in this study and the existing convex hull method are shown in Figure 23. Figure 23a is a route generated in the map space through the concave hull method proposed in this study, in which the distance moved was approximately 17.2 km, and the route generation time was 48 s. Figure 23b shows a route generated in the map space through the convex hull method, where the distance moved was approximately 21.9 km, and the route generation time was 61 s. The comparison results reveal that the route generated in the concave hull-based map space was approximately 5 km shorter, and the route generation time was approximately 13 s faster.

## 5. Conclusions

Route planning for ships is crucial not only for the safety of the ships but also for the safe passage of other ships at the same time and space. Therefore, for the safe route planning of a ship, sufficient safety must be secured from obstacles existing in the map space in which the route is searched. In particular, to generate the optimal path of the vessel within the coastal water, sufficient space that considers the underwater impediments must be ensured beyond the impediments on the water surface. Differentiating the non-navigable path, where risk factors lie, and determining the safety distance sufficiently beyond the water prohibited for sailing would be the greatest factors to consider. Therefore, in this study, a safety margin was defined based on the physical topographic space existing on the sea, the control space according to the characteristics of the ship, and the psychological space elements of the ship operator. In addition, we proposed an obstacle modeling method within the map space to ensure safety from obstacles at sea without degrading the efficiency of the route.

Water prohibited for sailing was established based on the safe water depths of the vessel on the electronic hydrograph image, and the hydrograph image underwent binarization for constituting the map area. This study defined prohibited water for sailing as an impediment to avoid during route searching of the vessel and the c-obstacle that expanded the size of the impediment as much as the safety distance, based on the safety area theory of the vessel navigator and the features of vessel control. Moreover, the concave hull method was used to minimize the area generated by impediments as a method of simplifying the boundary of the impediment. Finally, to validate the map space proposed by this study, the map space was created for certain model vessels within the island region of the southern sea of South Korea, and the PRM algorithm-based route-generation simulation was performed. The simulation results revealed that in map spaces in which the safety margin does not apply, routes that pass low-water depth areas or that are highly near the land were generated. In contrast, the case of applying safety margins ensured marginal areas in the generated routes; furthermore, the conventional convex hull method used in map composition research was compared with the proposed method in this study to verify its effectiveness. Simulation results showed that the area lost by vessels due to the impediments was established as a navigable area in the concave hull method. The PRM route generation results showed that the distance was reduced by approximately 5 km in the map space using the concave hull method, and the route generation time was approximately 13 s faster. The main contributions of this study are summarized as follows:

- The concepts of minimum safety margin through the UKC, the ship’s maneuvering characteristics, and bumper area were incorporated into the map space for path finding. It was confirmed that a route with minimum safety from obstacles was created.
- The concave hull algorithm was applied to simplify the boundary in the obstacle configuration space. It was confirmed that the loss area was minimized in the boundary configuration of obstacles; furthermore, it was possible to perform an efficient search in path finding, compared with the convex hull algorithm.

This study established the safety margin through vessel length, maximum draught, safety contour, the ship’s turning circle, and the ship’s domain. However, to generate the vessel route, the climate, knowledge of the marine environment, information for sailing within waters, and sailing regulations exist. Therefore, a real-time map-space-creation method is required in the future, considering various safety factors and optimal route information exchange with the vessel traffic service operator and the vessel navigator in vast coastal marine environments. Additionally, this study has some limitations, which are as follows:

- As the range of the target sea area increases, it may not be possible to express the detailed shape of the obstacle area when creating the map space.
- Because only vessels in a specific coastal area were included, the results may not be extensible to other geographical locations.

Nonetheless, in this paper, the obstacle modeling method was proposed through the concept of a safety margin to secure safety from obstacles in the sea without reducing the efficiency of the route, and it is expected to be able to help plan the safest route in coastal waters.

## Figures and Tables

**Table 1:** A summary of a literature review related to map representation for path finding.

Author (Year)	Achievement	Map Representation Method		Approach
		Classification	Considering Factor	
Lee et al. [6] (2019)	Shortest route on coastal	Cell decomposition	▪ Water depth ▪ Fuel consumption	▪ A*
Ari et al. [7] (2013)	Shortest route on coastal	Cell decomposition	▪ Ship’s Turning radius	▪ A
Ozkan et al. [8] (2019)	Composition of obstacle	Roadmap	▪ Aerial image ▪ Obstacles	▪ A*, GA, PRM ▪ Image processing
Lee et al. [9] (2017)	Shortest route on coastal	Cell decomposition Roadmap	▪ Coastline ▪ Weather data	▪ A* ▪ Quadtree ▪ Visibility graph
Shi et al. [10] (2018)	Composition of obstacle	Roadmap	▪ Satellite image ▪ Obstacle boundaries	▪ Dijkstra ▪ Image processing ▪ Convex hull
Masaudi [11] (2017)	Reduction of calculation time	Roadmap	▪ Obstacles boundary ▪ Points	▪ A*, Dijkstra ▪ Convex hull
Kim and Park [12] (2010)	Composition of obstacle	Roadmap	▪ Obstacles boundary ▪ Points	▪ Convex hull
Lim et al. [13] (2019)	Reduction of calculation time	Roadmap	▪ 3D Obstacles ▪ Contour line	▪ Visibility graph ▪ Convex hull

**Table 2:** PIANC’s depth components for the concept design (cd ) of ship-related factors.

Description	Speed	Wave	Outer Channel	Bottom
Depth (h)	All	Low swell $\left(H_{s}<1\;m\right)$	1.15 T to 1.2 T	Mud: None Sand/Clay: 0.5 m Rock/Coral: 1.0 m
		Moderate swell $\left(1\;m<H_{s}<1\;m\right)$	1.2 T to 1.3 T	
		Heavy swell $\left(H_{s}>2\;m\right)$	1.3 T to 1.4 T	

Assumes T>10 m. If T<10 m, use value for T=10 m (T means Draught of ship).

## Data Availability

Not applicable.
